# Molecular Detection of the Harmful Raphidophyte *Chattonella subsalsa* Biecheler by Whole-Cell Fluorescence *in-situ* Hybridisation Assay

**DOI:** 10.21315/tlsr2023.34.1.7

**Published:** 2023-03-31

**Authors:** Winnie Lik Sing Lau, Sing Tung Teng, Hong Chang Lim, Kieng Soon Hii, Sandric Chee Yew Leong, Chui Pin Leaw, Po Teen Lim

**Affiliations:** 1 Bachok Marine Research Station, Institute of Ocean and Earth Sciences, University of Malaya, 16310 Bachok, Kelantan, Malaysia; 2 Faculty of Resource Science and Technology, Universiti Malaysia Sarawak, 94300 Kota Samarahan, Sarawak, Malaysia; 3 Institute of Biodiversity and Environmental Conservation, Universiti Malaysia Sarawak, 94300 Kota Samarahan, Sarawak, Malaysia; 4 St. John’s Island National Marine Laboratory, Tropical Marine Science Institute, National University of Singapore, Singapore 119227

**Keywords:** *Chattonella*, harmful algal bloom, oligonucleotide probe, ribosomal DNA, fluorescence *in situ* hybridisation (FISH), *Chattonella*, kembangan alga yang berbahaya, oligonukleotid prob, DNA ribosom, penghibridan *in situ* berpendarfluor

## Abstract

Species of the genus *Chattonella (Raphidophyceae)* are a group of marine protists that are commonly found in coastal waters. Some are known as harmful microalgae that form noxious blooms and cause massive fish mortality in finfish aquaculture. In Malaysia, blooms of *Chattonella* have been recorded since the 1980s in the Johor Strait. In this study, two strains of *Chattonella* were established from the strait, and morphological examination revealed characteristics resembling *Chattonella subsalsa*. The molecular characterization further confirmed the species’ identity as *C*. *subsalsa*. To precisely detect the cells of *C. subsalsa* in the environment, a whole-cell fluorescence *in-situ* hybridisation (FISH) assay was developed. The species-specific oligonucleotide probes were designed *in silico* based on the nucleotide sequences of the large subunit (LSU) and internal transcribed spacer 2 (ITS2) of the ribosomal DNA (rDNA). The best candidate signature regions in the LSU-rRNA and ITS2-rDNA were selected based on hybridisation efficiency and probe parameters. The probes were synthesised as biotinylated probes and tested by tyramide signal amplification with FISH (FISH-TSA). The results showed the specificity of the probes toward the target cells. FISH-TSA has been proven to be a potential tool in the detection of harmful algae in the environment and could be applied to the harmful algal monitoring program.

## Abstract (fr)

Spesies genus Chattonella (Raphidophyceae) ialah sekumpulan protista marin yang biasa ditemui di perairan laut pantai. Sesetengahnya dikenali sebagai mikroalga berbahaya yang membentuk ledakan alga berbahaya dan menyebabkan kematian ikan secara besar-besaran dalam akuakultur ikan sirip. Di Malaysia, ledakan alga Chattonella telah direkodkan sejak tahun 1980-an di Selat Johor. Dalam kajian ini, dua strain Chattonella telah didirikan dari selat, dan pemeriksaan morfologi mendedahkan ciri-ciri yang menyerupai *Chattonella subsalsa*. Pencirian molekul seterusnya mengesahkan identiti spesies sebagai *C. subsalsa*. Untuk mengesan dengan tepat sel-sel *C. subsalsa* di dalam persekitaran, ujian penghibridan in-situ berpendarfluor (FISH) ke atas sel keseluruhan telah dibangunkan. Prob oligonukleotida spesies telah direka secara spesifik secara siliko berdasarkan jujukan nukleotida subunit besar (LSU) dan spacer transkripsi dalaman 2 (ITS2) gen DNA ribosom (rDNA). Calon terbaik kawasan tanda dalam LSU-rRNA dan ITS2-rDNA telah dipilih berdasarkan kecekapan penghibridan dan parameter prob. Prob telah disintesis sebagai prob biotinilasi dan diuji dengan penguatan isyarat tyramide dengan FISH (FISH-TSA). Keputusan menunjukkan kekhususan prob ke atas sel sasaran. FISH-TSA telah terbukti sebagai alat yang berpotensi dalam pengesanan alga berbahaya di alam sekitar dan boleh digunakan untuk program pemantauan alga berbahaya.

**Highlights**
- Two strains of the harmful raphidophyte *Chattonella subsalsa* were established from in the Johor Strait.
- A whole-cell fluorescence *in situ* hybridisation (FISH) assay targeting *C*. *subsalsa* cells was developed based on the nucleotide sequences of the LSU rDNA and ITS2.
- The species-specific probes developed showed specificity toward the target cells, thus having the potential to detect this harmful microalga in the environment.

## INTRODUCTION

Harmful algal bloom (HAB), also known as “red tide”, occurs when harmful microalgae grow in high biomass in the water column, causing severe consequences such as food poisoning syndromes in humans who consume algal toxins-contaminated seafood and massive mortality of marine organisms (Hoagland *et al*. 2002). Paralytic shellfish poisoning has been the focus of attention in Malaysia, as it has been linked to the majority of human intoxication cases (Lim *et al*. 2012; Usup *et al*. 2012; Yñiguez *et al*. 2021). Several causative dinoflagellates, including Pryodinium bahamense Plate, Alexandrium tamiyavanichii Balech, A. minutum Halim, and Gymnodinium catenatum Graham, have been documented throughout the Malaysian waters (Leaw *et al*. 2005; Lim *et al*. 2007). Nonetheless, other algal-related incidents have been documented in Malaysia, such as massive fish kills in aquaculture farms (Lim *et al*. 2012; 2014; Teng *et al*. 2016; Yñiguez *et al*. 2021; Lum *et al*. 2021). The majority of these events have been linked to marine harmful dinoflagellates, such as *Margalefidinium polykrikoides* (Margalef) *Gómez*, Richlen, and Anderson, *Noctiluca scintillans* (Macartney), Kofoid & Swezy, and *Karlodinium australe* Salas, Bolch, and Hallegraeff (Lim *et al*. 2014; Teng *et al*. 2016).

Among the harmful microalgae, several groups of raphidophytes have been recognized as harmful to marine organisms (Lum *et al*. 2021). Members of the genus *Chattonella Biecheler* are among those that have caused severe damage to the aquaculture industries in many coastal countries, for instance Japan (Okaichi 2003; Imai & Yamaguchi 2012). The first record of *Chattonella* bloom has been reported on the Malabar Coast, India, while the most severe fish kill event was recorded in Harima-Nada, the Seto Inland Sea, Japan, in the summer of 1972 (Imai & Yamaguchi 2012). In Malaysia, the occurrence was first documented in 1983 along the Johor strait (Maclean 1989).

Conventionally, light microscopy has been used to identify the morphological characteristics of *Chattonella* species. The species are unicellular, bi-flagellated, and pigmented, ranging from golden brown to greenish in some species depending on the fucoxanthin content (Klöpper *et al*. 2013). In general, species of *Chattonella* are differentiated based on cell size, cell shape, presence of a hyaline posterior tail, and mucocysts (Hara & Chihara 1982; Hara *et al*. 1994; Bowers *et al*. 2006). However, diversity in the morphology of *Chattonella* is high, even within the same species. Often, molecular characterization using gene markers such as ribosomal RNA genes (rDNA) is required to aid species recognition (Bowers *et al*. 2006; Demura *et al*. 2009). Among the species of *Chattonella, C. antiqua (Hada) Ono, C. marina (Subrahmanyan) Hara* and *Chihara, C. ovata Hara* and *Chihara* (also referred to as *C. marina* complex sensu Demura *et al*. 2009), and *C. subsalsa Biecheler* have been reported to cause HABs that are associated with massive farmed-fish mortality and impact the economies of affected countries worldwide (Hiroishi *et al*. 2005; Edvardsen & Imai 2006; Imai *et al*. 2006; Imai & Yamaguchi 2012; Lum *et al*. 2021).

In the Johor Strait shared between Malaysia and Singapore, the occurrence of *Chattonella* has often been reported from the monitoring and research studies of both countries (Khoo & Wee 1997; Leong *et al*. 2015; Tan *et al*. 2016; Kok *et al*. 2019; Liow *et al*. 2019). Morphological plasticity in the species, however, has hampered precise species recognition, particularly in the preserved environmental samples, where the cells tend to deform and the morphology deteriorates after fixation. Morphological plasticity in the species, however, has hampered precise species recognition, particularly in the preserved environmental samples, where the cells tend to deform and the morphology deteriorates after fixation (Katano *et al*. 2009). This often leads to species misidentification. Alternative approaches, such as molecular techniques (Bower *et al*. 2006; Stacca *et al*. 2016), could therefore be explored to overcome the limitation. In this study, whole-cell tyramide signal amplification-fluorescence *in situ* hybridisation (FISH-TSA) was developed to detect the harmful raphidophyte *Chattonella subsalsa*. The ribosomal RNA-targeted species-specific probes were designed *in silico* and applied in the assay.

## MATERIALS AND METHODS

### Algal Cultures and Morphological Observation

Live plankton samples were collected from the Johor Strait using a 20 μm-mesh plankton net and vertically hauled into subsurface seawater (< 5 m) during high tide. The micropipette technique was used to isolate the targeted cells. Cultures were established and grown in f/2 medium. Live plankton samples were collected from the Johor Strait using a 20 μm-mesh plankton net and vertically hauled into subsurface seawater (< 5 m) during high tide. The micropipette technique was used to isolate the targeted cells. Cultures were established and grown in f/2 medium (Guillard & Ryther 1962) with a salinity of 30, 25 ± 0.5°C, under a light intensity of 100 μmol photons m^−2^ s^−1^, with a 12: 12 h light: dark photoperiod.

Morphological observation of cell shape and chloroplast was performed using an Olympus IX51 research microscope (Olympus, Tokyo, Japan). To observe the nuclear position, cells were first stained with the DAPI-nuclei stain and then examined under ultraviolet light with a UV filter set. Digital images were captured with an Olympus DP72 digital camera (Olympus, Tokyo, Japan).

### Genomic DNA Extraction, rDNA Amplification and Sequencing

The genomic DNA of *Chattonella* cultures was extracted as described by Leaw *et al*. (2010). In brief, the mid-exponential cells from 200 mL of cultures were harvested by centrifugation (1100 ×*g*, one min). The cell pellets were rinsed with ddH_2_O and resuspended in 10× NET lysis buffer (5 M NaCl, 0.5 M EDTA, 1 M Tris-HCl, pH 8) and 1% sodium dodecyl sulphate. The mixture was incubated at 65°C and subsequently extracted with chloroform: isoamyl alcohol (24:1) and phenol: chloroform: isoamyl alcohol (25:24:1). The genomic DNA was then precipitated by adding absolute ethanol and 3 M sodium acetate (pH 5). The DNA pellet was then rinsed with cold 70% ethanol. Finally, the DNA pellet was dissolved in 30 μL of TE buffer (10 mM Tris-HCl, pH 7.4; and 1 mM EDTA, pH 8) and stored at −20°C until further analysis.

The large subunit (LSU) rDNA was amplified using a pair of universal primers: D1R (5′-ACC CGC TGA ATT TAA GCA TA-3′) and D3Ca (5′-ACG AAC GAT TTG CAC GTC AG-3′) (Scholin *et al*. 1994); while the internal transcribed spacer (ITS) region was amplified using the primer pairs: ITSA (5′-GTA ACA AGG THT CCG TAG GT-3′), ITSB (5′-AKA TGC TTA ART TCA GCR GG-3′) (Adachi *et al*. 1994), or the primer pair, ITSFC (5′-TAG AGG AAG GTG AAG TCG-3′), ITSFR (5′-TTA CTA GGG GAA TCC GAG-3′) designed in this study. The 25 μL PCR mixtures contained 1× PCR buffer, 2 mM MgCl_2_, 0.02 μM of each primer, 0.2 mM of each dNTP, 2.5 U *Taq* polymerase (Invitrogen, Life Technologies, USA), and 20 ng μL^−1^ −100 ng μL^−1^ of genomic DNA. The amplification was performed by using an Artik 5020 thermal cycler (ThermoScientific, USA). The amplicons were further purified by the QIA quick purification kit (QIAGEN, Germany, Hilten), and single-pass DNA sequencing was performed on an ABI 3700XL automated DNA sequencer (Applied Biosystems, USA), with both strands sequenced.

### Phylogenetic Analyses

Taxon sampling was performed by retrieving the LSU and ITS-rDNA nucleotide sequences of *Chattonella* species from the NCBI GenBank nucleotide database (Table 1). The sequences of *Heterosigma akashiwo* were used as an outgroup. The newly obtained *C. subsalsa* sequences in this study and the retrieved sequences were multiple aligned using the program MUSCLE (https://www.ebi.ac.uk/Tools/msa/muscle/). The aligned datasets were phylogenetic inferred using Phylogenetic Analysis Using Parsimony* (PAUP*) v4.0 b10 (Swofford 2003) and MrBayes v3.1.2 (Huelsenbeck & Ronquist 2001), as described in Leaw *et al*. (2016).

### *In silico* rRNA-Targeted Oligonucleotide Probe Design

The rDNA sequences of *Chattonella* species retrieved from GenBank and SILVA (http://www.arb-silva.de/) public databases were used to identify potential signature regions by using the PROBE_DESIGN tool of the ARB programme package (Ludwig *et al*. 2004). The parameters for probe design included probe length, percentage of GC content, melting temperature (T_m_), and self-complementary (Kumar *et al*. 2005). The probe candidates were selected for both target and probe sequences and were displayed in a result list (Kumar *et al*. 2005; Tables 2 and 3). The selected probe candidates were then evaluated using the PROBE Match Tool (PMT) of the ARB. The oligonucleotide sequences were then subjected to extensive specificity tests through BLAST comparisons against nucleotide databases of non-target sequences. The candidate sequences that complemented the region of target sequences with at least one mismatch in other non-target sequences were chosen (Hugenholtz *et al*. 2002). BLAST was also used to confirm that the sequences were transcribed in the correct orientation (Hugenholtz *et al*. 2002). The selected probes satisfying the *in silico* experimental constraints were then synthesised as a biotinylated probe (IDT Inc., Singapore).

### Tyramide Signal Amplification-fluorescence *in situ* Hybridisation

Cells were fixed with Lugol iodine solution (~1%) and transferred to a glass slide that was pre-fixed with 2% HistoGrip^TM^ (Invitrogen, Life Technologies, USA) (Breininger & Baskin 2000). The fixed cells were air-dried and later rinsed twice with 5× SET hybridisation buffer (10% Nonidet) and allowed to stand in the buffer for 3 min (Chen *et al*. 2008). Then, the probe was added to the slide containing the cells. The slide was incubated in a dry bath at 58°C for 30 min. The slide was washed twice with a 5 SET buffer after incubation.

Following that, 1% blocking reagent was added and incubated at room temperature for 30 min, horseradish peroxidase (HRP) solution was added to the slide and incubated at room temperature for 30 min. The glass slide was then washed with phosphate buffered saline (PBS) that was pre-heated to 37°C. The tyramide working solution (TSA kit with Alexa Fluor® 488 Tyramide; Molecular Probe®, Life Technologies, USA) was then added to the slide in the dark and incubated at room temperature for 10 min. The slide was rinsed again in PBS to remove any excess tyramide working solution. The universal UniC probe (positive control) (5′-/5Biosg/ GWA TTA CCG CGG CKG CTG-3′) and UniR probe (negative control) (5′-/5Biosg/ CAG CMG CCG CGG TAA TWG-3′) were used as controls (Lebaron *et al*. 1997).

The slides were then observed under an Olympus IX51 microscope equipped with a filter set (470 nm–490 nm excitation and 510 nm–550 nm emission) under UV light. Digital images were captured with an Olympus DP72 digital camera (Olympus).

## RESULTS

### Species Identification

**Figure 1:**
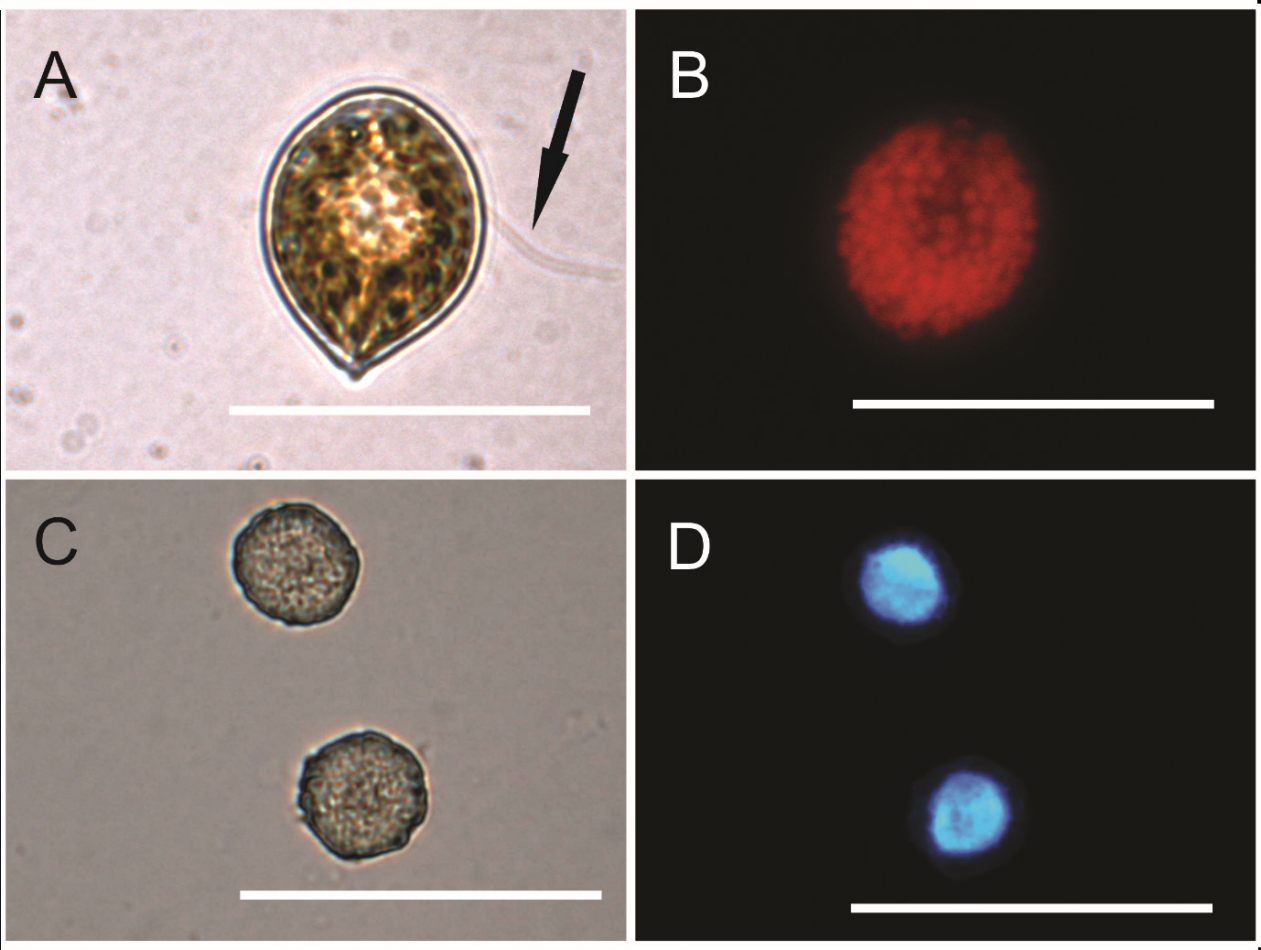
Light and epi-fluorescence micrographs of Chattonella subsalsa. (A) Flagellum-containing cell (arrow); (B) An autofluorescence micrograph of a cell showing the chloroplast; (C–D) Cells with DAPI-stained nuclei show the position of nuclei. The scale is 50 μm.

Two strains of *C. subsalsa* from the Johor Strait were established and used in this study. Cells of the two strains showed similar morphology, with cell dimensions of 36.6 ± 2.9 μm long and 20.5 ± 4.5 μm wide (*n* = 50). Under LM, cells are oval, pear-like in shape, which is similar to other *C. subsalsa* reported previously (Fig. 1). There are two sub-equal, hetero-dynamic flagella at the anterior of the cells (Fig. 1A). The flagella can only be observed in the living cells. The cells contain many golden-brown chloroplasts, which appear barrel-shaped (Fig. 1B). The nucleus is large and appears oval in shape; it is located in the middle of the cell (Fig. 1D).

### Phylogenetic Inferences of LSU and ITS rDNA

**Figure 2:**
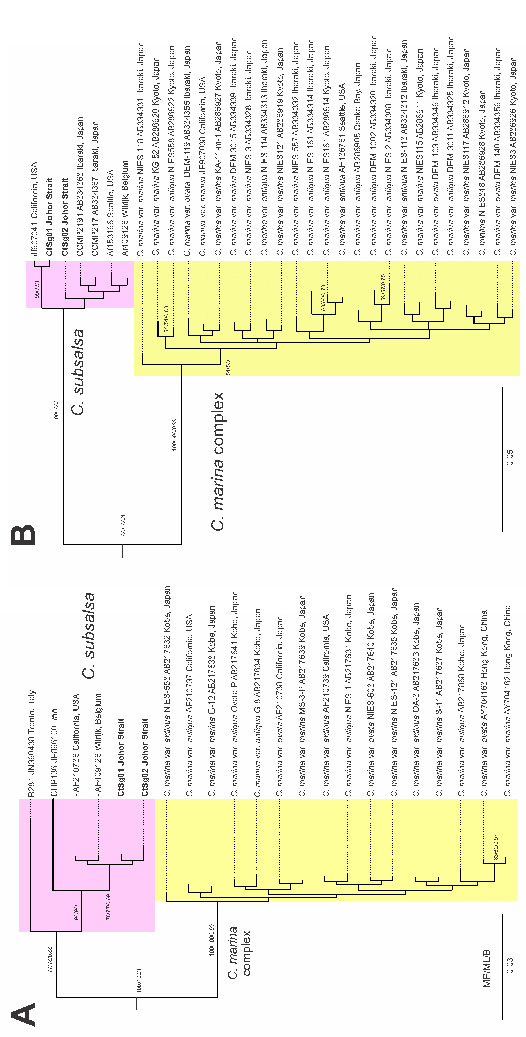
The Bayesian inference of the LSU rDNA dataset (A) and ITS dataset (B) of *Chattonella* species. Outgroups were not shown. The strains of *C*. *subsalsa* obtained in this study are in boldface.

A total of 23 LSU rDNA sequences and 30 sequences of the ITS of *Chattonella* were retrieved from the GenBank nucleotide database. Both LSU and ITS rDNA datasets yielded identical tree topologies for maximum parsimony (MP), maximum likelihood (ML), and Bayesian inference (BI); the BI tree is shown in Fig. 2. The trees revealed two monophyletic clades with strong support values (MP/ML/BI, 100/100/1); one clade comprised species in the *C*. *marina* complex: *C. marina* var. *antiqua, C. marina* var. *marina, C. minima*, and *C. marina* var. *ovata*, while the other clade comprised only taxa from *C. subsalsa*. The two *C. subsalsa* strains (CtSg01 and CtSg02) in this study were grouped with other *C. subsalsa* strains and formed a distinct clade that separated from the strains of *C. marina* complex, according to both LSU and ITS phylogenetic trees.

### Species-Specific Oligonucleotide Probes of *Chattonella subsalsa*

#### LSU rRNA signature region and probe

In the first run, a total of 21 candidate sequences of the potential signature regions in the LSU rDNA of *C. subsalsa* were detected at a 730-nucleotide length (Table 2). At least one mismatch was found between the related species, such as *C. marina* var. *antiqua* and *C. marina*. The probes selected *in silico* by ARB contained 18 bases, with GC contents in the range of 50% to 70%. Several of them showed the Gibb energy (ΔG°) greater than −14 kcal/mol, indicative of secondary structure formation (Table 2). A confirmatory test of the probe specificity was performed by blasting in the nucleotide database. The blastn results showed that the probes selected were not specific to *C. subsalsa*, where the probes matched diatom species with 100% coverage and 100% identity.

**Figure 3:**
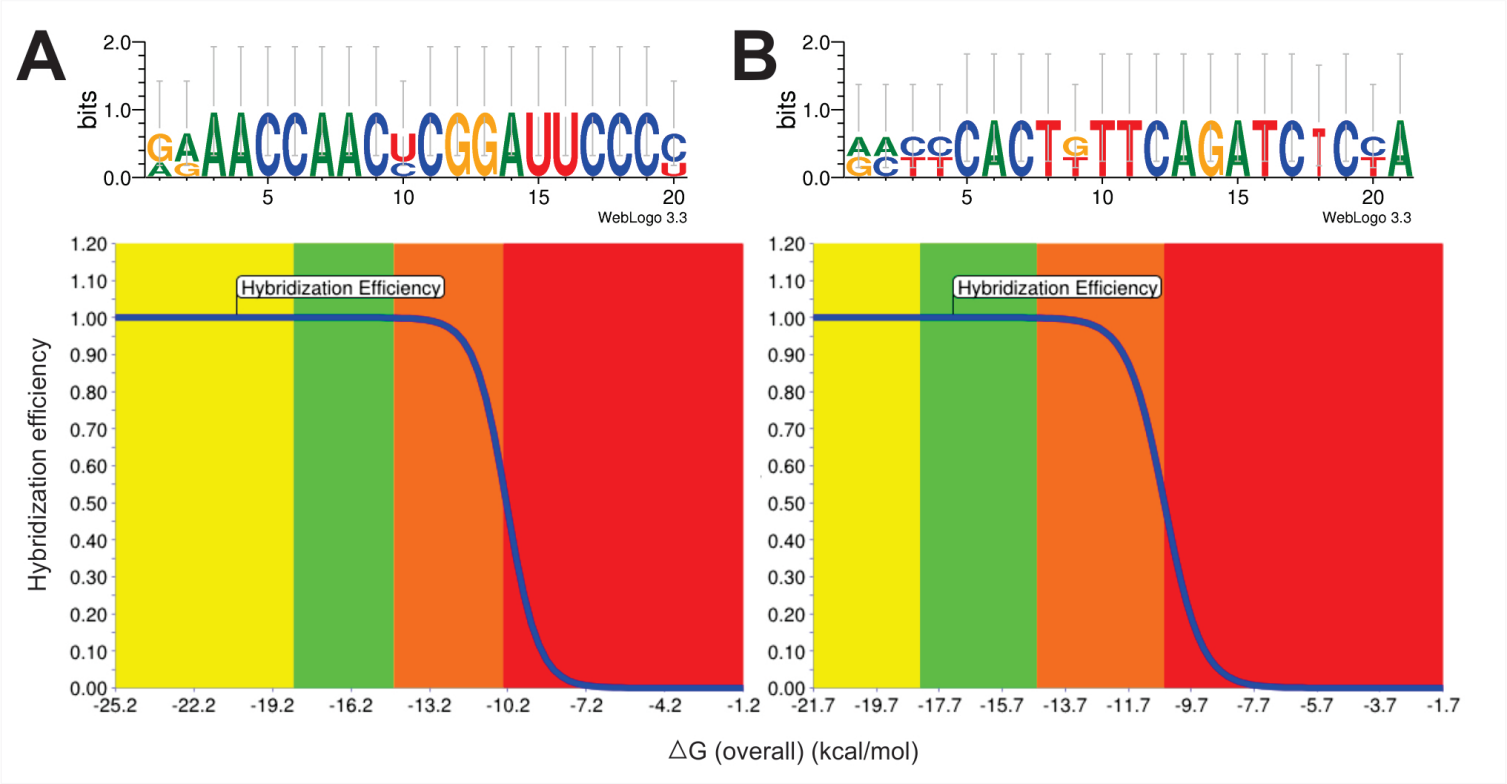
Signature sequences of *Chattonella subsalsa* identified in this study (sequence direction, 5′→3′), (A) L-S-C.sub-0039-a-A-20, and (B) L-S-C.sub-0219-a-A-21; and their hybridisation efficiency curves.

Therefore, a second attempt at *in silico* analysis was performed with a slight modification of the signature regions. A total of seven candidate sequences were chosen (Table 3). The length of the probes was in the range of 19 bases to 23 bases, longer than the first run, to ensure the presence of GC complementary pairs at the start and end of the probe sequences. Subsequently, the parameters of the probes were determined, and the specificity of the probes was evaluated through blastn search. Out of the seven probe candidates, Probe Set 7 (5′-GGG GAA UCC GGG UUG GUU UC-3′) was selected (Fig. 3) based on its high GC content (60%), lowest Gibb energy (ΔG° = −20.2 kcal/mol), and lower melting point (58.6°C) in contrast to other probes (Table 3). The sequence was further synthesised as a biotinylated probe to perform the FISH assay in the later analysis. According to the Probe Nomenclature, the probe was designated as L-S-C.sub-0039-a-A-20 (Alm *et al*. 1996).

#### ITS2 rRNA signature region and probe

The ITS2 region of the rDNA was used to design a species-specific probe as it is more specific at the species level than the LSU rDNA. In this study, ten candidate sequences of *C*. *subsalsa* were determined from a 262-bp-long ITS2-rDNA complete sequence; the sequences that are expected to identify the target are listed in Table 4. The candidate sequence length was in the range of 18 bases to 21 bases. These candidate sequences were then subjected to specificity analysis by performing BLAST comparisons against the nucleotide databases, and the results showed that there was no match to other non-target species. Among the ten candidate sequences (Table 4), probe set 10 (5′-TGG AGA TCT GAA CAG TGA GG-3′) was chosen because it exhibited a lower ΔG°, which is −16.7 kcal/mol, comprised of 52.4% of the GC pair with a 100% hybridisation efficiency. Most importantly, the probe is unique to *C. subsalsa*, and a total of six mismatches were found in the sequence when compared to other non-target species (Fig. 3B). This ITS2 probe was designated as I-S-C.sub-0219-a-A-21 and synthesised as a biotinylated probe for later hybridisation experiments.

#### Tyramide signal amplification-fluorescence in situ hybridisation (FISH-TSA)

**Figure 4:**
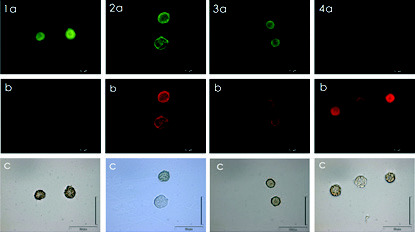
Micrographs of *Chattonella subsalsa *cells treated with UniC positive control (1a), LSU-rRNA probe (2a), ITS2-rDNA probe (3a), and UniR negative control (4a). LM (1c–4c), cells with chloroplast autofl uorescence (1b–4b).

**Figure 5:**
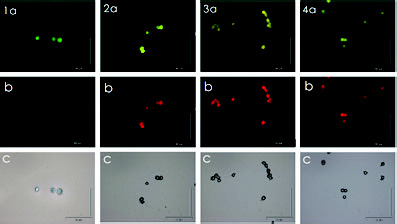
Micrographs of *Heterosigma akashiwo *cells treated with UniC positive control (1a), LSU-rRNA probe (2a), ITS2-rDNA probe (3a), and UniR negative control (4a). LM (1c–4c), cells with chloroplast autofl uorescence (1b–4b).

The FISH-TSA assay with the biotinylated-labelled probes was tested on the clonal cultures of *C. subsalsa*. The species *Heterosigma akashiwo* was used as the non-target species. When treated with the positive-control eukaryotic-universal UniC probe, the hybridised cells of *C. subsalsa* and *H. akashiwo* showed bright green fluorescence signals (Fig. 4). Lime-green fluorescence signals were observed when *C. subsalsa* cells were hybridised with *C. subsalsa* LSU-rRNA and ITS-rDNA species-specific probes (Fig. 4). In contrast, when the cells were treated with the negative-control UniR probe, they showed chartreuse-yellow fluorescence with low intensity (Fig. 4). When the *C. subsalsa* species-specific probes were tested on *H. akashiwo* cells, chartreuse-yellow fluorescent signals were observed, indicating negative results (Fig. 5).

## DISCUSSION

In this study, two species-specific oligonucleotide probes in the LSU-rRNA and ITS2-rDNA were developed to detect the harmful raphidophyte *Chattonella subsalsa*. The probes were applied in the assay of whole-cell fluorescence *in situ* hybridisation (FISH) for species detection. The region of the LSU-rRNA gene was chosen to owe to have a universally conserved region while exhibiting some taxon-specific variable regions (Amann & Ludwig 2000). However, the results of the specificity analysis on the LSU-rRNA selected sequences showed cross-identity with other *Chattonella* species and diatom species. Therefore, a more taxon-specific rDNA region, the ITS2-rDNA, has been selected to design the species-specific probe of *C*. *subsalsa*.

The biotinylated probes developed in this study have been tested on the *C. subsalsa* cells through the assay of FISH-TSA. The technique of FISH has been widely used in identifying HAB species such as *Pseudo-nitzschia* spp., *Alexandrium* spp., and *Karenia brevis* (Davis) Hansen & Moestrup (Miller & Scholin 1998, Chen *et al*. 2008). The method, however, has been shown to exhibit less sensitivity when observed under an epi-fluorescence microscope (Lecuyer *et al*. 2008). The efficiency of FISH, therefore, has been improved by tyramide signal amplification (TSA) to obtain a better resolution in the FISH application (Lecuyer *et al*. 2008). FISH-TSA is a protocol that enables detection with a very small probe by signal amplification (Schriml *et al*. 1999). The biotinylated probes have been designed to achieve the enzymatic action of HRP as they provided strong enzymatically amplified signals and improved the resolution (Kerstens *et al*. 1995).

In this study, both LSU-rRNA and ITS2-rDNA probes of *C. subsalsa* exhibited positive green fluorescent signals when hybridised into the cells of *C. subsalsa*. Generally, the ITS2-rDNA probe does not give whole-cell fluorescence as it was only hybridised to the nucleus of the cells. However, cells of *C. subsalsa* that were applied with the ITS2-rDNA probe showed almost whole-cell fluorescence owing to its large nucleus as shown in Fig. 1.

To confirm the specificity of the probes, both *C. subsalsa* species-specific probes were tested with the non-target species *H. akashiwo*. The results showed that *H. akashiwo* showed light-yellow fluorescence when tested with the ITS2-rDNA probe, like the negative control. This showed that the ITS2-rDNA probe was specific only to *C*. *subsalsa*. But when tested with the LSU-rRNA probe, it showed yellow-green fluorescence that made it difficult to evaluate if the result was positive or negative. It is thus suggested that the ITS2-rDNA probe is better than the LSU-rRNA probe in detecting *C*. *subsalsa*.

The assay of FISH-TSA was applied to microscope glass slides throughout the study. This method has been previously described by Chen *et al*. (2008) as applied to *H. akashiwo* cells. The cell harvesting procedures such as centrifugation and filtration that were previously applied to the armoured dinophyte *Alexandrium* and the diatom *Pseudo-nitzschia* (Miller & Scholin 1998) were less suitable in this case as cells tend to burst when undergoing centrifugation or filtration.

Several factors affect the efficiency of FISH-TSA. Physiological growth conditions of the cells are among the factors that affect FISH-TSA detection (Sako *et al*. 2004, Chen *et al*. 2008). Kim *et al*. (2004) discovered that exponentially growing cells have higher fluorescent intensities than stationary phase cells. The low fluorescent intensity of the cells was likely due to the decreasing rRNA content in stationary-phase cells (Anderson *et al*. 1999).

## CONCLUSION

To conclude, the species-specific oligonucleotide probe of *C. subsalsa* was successfully designed in the ITS2-rDNA region. The results of this study revealed that the ITS2 probe was more specific as compared to the LSU probe. The strong fluorescent signal in FISH-TSA also proves its efficiency in detecting harmful algal species from environmental samples. Future field applications should be carried out to further evaluate the feasibility of this assay for HAB monitoring purposes.

## Figures and Tables

**Table 1:** LSU-rDNA (D1–D3) and ITS sequences of *Chattonella* species that were used in this study, with strain, location, GenBank accessions, and references.

Species	Strain	LSU Accession	ITS Accession	Location	References
*C. marina* var.*antiqua*	–	–	AF136761	Seattle, USA	Connell 1999
	–	AF210737	–	California, USA	Tyrrell *et al*. 1999*
	–	AB217868	–	Kobe, Japan	Tanabe *et al*. 2005*
	NIES–558	AB217632	–	Kobe, Japan	Tanabe *et al*. 2005
	Ovata–P	AB217641	–	Kobe, Japan	Tanabe *et al*. 2005*
	NIES–1	AB217631	–	Kobe, Japan	Tanabe *et al*. 2005*
	G–8	AB217634	–	Kobe, Japan	Tanabe *et al*. 2005*
	OA–3	AB217633	–	Kobe, Japan	Tanabe *et al*. 2005*
	NIES558	–	AB286922	Kyoto, Japan	Kamikawa *et al*. 2007
	NIES161	–	AB286914	Kyoto, Japan	Kamikawa *et al*. 2007
	NIES–161	–	AB334314	Ibaraki, Japan	Demura & Kawachi 2007*
	NIES–114	–	AB334313	Ibaraki, Japan	Demura & Kawachi 2007*
	NIES–113	–	AB334312	Ibaraki, Japan	Demura & Kawachi 2007*
	NIES–2	–	AB334308	Ibaraki, Japan	Demura & Kawachi 2007*
	–	–	AB286905	Osaka Bay, Japan	Kamikawa *et al*. 2007
	DEM–3011	–	AB334325	Ibaraki, Japan	Demura & Kawachi 2007*
	DEM–1002	–	AB334320	Ibaraki, Japan	Demura & Kawachi 2007*
*C. marina*	KG–52	–	AB286920	Kyoto, Japan	Kamikawa *et al*. 2007
	–	AF210739	–	California, USA	Tyrrell *et al*. 1999*
	MS–3–P	AB217639	–	Kobe, Japan	Tanabe *et al*. 2005*
	G–12	AB217638	–	Kobe, Japan	Tanabe *et al*. 2005*
	S–11	AB217637	–	Kobe, Japan	Tanabe *et al*. 2005*
	KA–11–m–1	–	AB286927	Kyoto, Japan	Kamikawa *et al*. 2007
	NIES3	–	AB286926	Kyoto, Japan	Kamikawa *et al*. 2007
	NIES121	–	AB286919	Kyoto, Japan	Kamikawa *et al*. 2007
	NIES117	–	AB286912	Kyoto, Japan	Kamikawa *et al*. 2007
	NIES115	–	AB286911	Kyoto, Japan	Kamikawa *et al*. 2007
	DEM–3015	–	AB334339	Ibaraki, Japan	Demura & Kawachi 2007*
	NIES–557	–	AB334332	Ibaraki, Japan	Demura & Kawachi 2007*
	NIES–118	–	AB334331	Ibaraki, Japan	Demura & Kawachi 2007*
	NIES–3	–	AB334328	Ibaraki, Japan	Demura & Kawachi 2007*
	NIES–121	AB217635	–	Kobe, Japan	Tanabe *et al*. 2005*
	–	AY704162	–	Hong Kong, China	Cheung *et al*. 2004*
	S–11	AB217637	–	Kobe, Japan	Tanabe *et al*. 2005*
	–	–	JF907038	California, USA	Band-Schmidt *et al*. 2011*
*C. minima*	NIES848	–	AB286928	Kyoto, Japan	Kamikawa *et al*. 2007
*C. marina* var. *ovata*	DEM–140	–	AB334359	Ibaraki, Japan	Demura & Kawachi 2007*
	NIES–603	AB217640	–	Kobe, Japan	Tanabe *et al*. 2005
	DEM–119	–	AB334355	Ibaraki, Japan	Demura & Kawachi 2007*
	DEM–103	–	AB334349	Ibaraki, Japan	Demura & Kawachi 2007*
	–	AF210738	–	California, USA	Tyrrell *et al*. 1999*
*C. marina* var. *ovata*	–	AY704163	–	Hong Kong, China	Cheung *et al*. 2004*
*C*. sp. R281	–	JN390438	–	Trento, Italy	D’Alelio 2011*
*C. subsalsa*	–		AF153196	Seattle, USA	Connell 2000
	–	AF409126	AF409126	Wilrijk, Belgium	Ben Ali *et al*. 2002
	–	AF210736	–	California, USA	Tyrrell *et al*. 1999*
	CCMP217	–	AB334367	Ibaraki, Japan	Demura & Kawachi 2007*
	–	–	JF907041	California, USA	Band-Schmidt *et al*. 2011*
	CCMP2191	–	AB334368	Ibaraki, Japan	Demura & Kawachi 2007*

*Note*:

* Sequence with direct submission

**Table 2:** First *in silico* selection of the LSU-rRNA probe signature regions of *Chattonella subsalsa*, with probe length constraint to 18 bases and their parameters.

Set	Target sequence (5′–3′)	Probe sequence (5′–3′)	GC content (%)	ΔG° (kcal/mol)	Hybridisation efficiency	T_m_ (°C)	Position in alignment (5′–3′)
1	CUUGAAACACGGGACCAA	UUGGUCCCGUGUUUCAAG	50	−7.2	0.0376	52.8	711–728
2	GUCUUGAAACACGGGACC	GGUCCCGUGUUUCAAGAC	55.6	−11.4	0.9697	53.4	709–726
3	UCUUGAAACACGGGACCA	UGGUCCCGUGUUUCAAGA	50	−14	0.9995	53.8	710–727
4	UGAAACACGGGACCAAGG	CCUUGGUCCCGUGUUUCA	55.6	−17.3	1	55.1	713–730
5	UUGAAACACGGGACCAAG	CUUGGUCCCGUGUUUCAA	50	−14.5	0.9998	52.8	712–729
6	CGUCUUGAAACACGGGAC	GUCCCGUGUUUCAAGACG	55.6	−13.4	0.9987	50.9	708–725
7	CCGUCUUGAAACACGGGA	UCCCGUGUUUCAAGACGG	55.6	−12.3	0.9928	55	707–724
8	CCCGUCUUGAAACACGGG	CCCGUGUUUCAAGACGGG	61.1	−11.9	0.9853	56.3	706–723
9	GAAAAGAAACCAACCCGG	CCGGGUUGGUUUCUUUUC	50	−18.1	1	51.5	34–51
10	GGAAAAGAAACCAACCCG	CGGGUUGGUUUCUUUUCC	50	−17.2	1	51.5	33–50
11	AAACCAACCCGGAUUCCC	GGGAAUCCGGGUUGGUUU	55.6	−17.1	1	55.4	40–57
12	AACCAACCCGGAUUCCCC	GGGGAAUCCGGGUUGGUU	61.1	−18.1	1	57.8	41–58
13	AACCCGGAUUCCCCUAGU	ACUAGGGGAAUCCGGGUU	55.6	−16.1	1	55.3	45–62
14	ACCAACCCGGAUUCCCCU	AGGGGAAUCCGGGUUGGU	61.1	−18	1	59	42–59
15	ACCCGGAUUCCCCUAGUA	UACUAGGGGAAUCCGGGU	55.6	−15.3	0.9999	54.4	46–63
16	AGAAACCAACCCGGAUUC	GAAUCCGGGUUGGUUUCU	50	−15.9	1	52.3	38–55
17	CAACCCGGAUUCCCCUAG	CUAGGGGAAUCCGGGUUG	61.1	−16.3	1	54.9	44–61
18	CCAACCCGGAUUCCCCUA	UAGGGGAAUCCGGGUUGG	61.1	−16.6	1	56.6	43–60
19	CCCGGAUUCCCCUAGUAA	UUACUAGGGGAAUCCGGG	55.6	−13	0.9976	53	47–64
20	CCGGAUUCCCCUAGUAAC	GUUACUAGGGGAAUCCGG	55.6	−13.1	0.9978	51.6	48–65
21	GAAACCAACCCGGAUUCC	GGAAUCCGGGUUGGUUUC	55.6	−16.2	1	53.6	39–56

**Table 3:** Second *in silico* selection of the LSU-rRNA probe signature regions of *Chattonella subsalsa* and their parameters.

Set	Target Sequence (5′–3′)	Probe Sequence (5′–3′)	GC Content (%)	ΔG° (kcal/mol)	Hybridisation efficiency	T_m_ (°C)	Length (bp)	Position in alignment (5′–3′)
1	CCAACCCGGAUUCCCCUAG	CUAGGGGAAUCCGGGUUGG	63.2	−18.4	1	57.4	19	43–61
2	GAACCAACCCGGAUUCCCC	GGGGAAUCCGGGUUGGUUC	63.2	−19	1	58.5	19	39–59
3	GAAAAGAAACCAACCCGGAUUC	GAAUCCGGGUUGGUUUCUUUUC	45.5	−19.7	1	54.5	22	34–55
4	CCAACCCGGAUUCCCCUAGTAAC	GUUACUAGGGGAAUCCGGGUUGG	56.5	−20.2	1	59.1	23	43–65
5	CCAACCCGGAUUCCCCUA	UAGGGGAAUCCGGGUUGG	61.1	−16.6	1	56.6	18	43–60
6	CCCGGAUUCCCCUAGUAACGG	CCGUUACUAGGGGAAUCCGGG	61.9	−19	1	59.1	21	47–67
**7**	GAAACCAACCCGGAUUCCCC	GGGGAAUCCGGGUUGGUUUC	60	−20.2	1	58.6	20	39–58

**Table 4:** *In silico* selection of the ITS2-rDNA probe signature regions of *Chattonella subsalsa* and their parameters.

Set	Target sequence (5′–3′)	Probe sequence (5′–3′)	GC (%)	ΔG° (kcal/mol)	Hybridisation efficiency	T_m_ (°C)	Length (bp)	Position in alignment (5′–3′)
1	CCGCCTCACTGTTCAGAT	ATCTGAACAGTGAGGCGG	55.6	−8.2	0.0864	54.6	18	217–234
2	CCTCACTGTTCAGATCTC	GAGATCTGAACAGTGAGG	50	−12.3	0.985	49.1	18	220–237
3	CGCCTCACTGTTCAGATC	GATCTGAACAGTGAGGCG	55.6	−9.6	0.4953	52.9	18	218–235
4	CTCACTGTTCAGATCTCC	GGAGATCTGAACAGTGAC	50	−12	0.9773	49.4	18	221–238
5	GCCTCACTGTTCAGATCT	AGATCTGAACAGTGAGGC	50	−12.8	0.9934	51.4	18	219–236
6	GGTGGCTCTGCCGCCTCACT	AGTGAGGCGGCAGAGCCACC	70	−13.8	0.9985	65.0	20	207–226
7	GTGGCTCTGCCGCCTCACTG	CAGTGAGGCGGCAGAGCCAC	70	−12.7	0.992	63.5	20	208–227
8	TGGCTCTGCCGCCTCACTGT	ACAGTGAGGCGGCAGAGCCA	65	−13.9	0.9987	64.1	20	209–228
9	CCGCCTCACTGTTCAGATCTC	GAGATCTGAACAGTGAGGCGG	57.1	−11.5	0.9443	57.4	21	217–237
10	GCCTCACTGTTCAGATCTCCA	TGGAGATCTGAACAGTGAGGC	52.4	−16.7	1	56.5	21	219–239
